# On the Pathology of Pulmonary Tuberculosis

**Published:** 1872-09-15

**Authors:** 


### Original Communications.

### ON THE PATHOLOGY OF PULMONARY TUBERCULOSIS.

[Read before the Chicago Medical Society September 16, 1872, by S. R. Millard, M. D.]

Gentlemen: It is with the deepest feelings of my own inability to handle, as well as a just appreciation of the magnitude of, the subject, that I approach the so-much-avoided yet familiar disease of pulmonary tuberculosis, knowing as I do that in a dissertation of the present character an attempt to describe its true pathology and philosophical treatment must of necessity be brief and imperfect. It is a fact that we daily walk amid scenes of a sublime and mysterious nature, without our souls being moved by their influences, or our minds awakened to their investigation. We find in this the reason. We have been accustomed to them from childhood, and that which is always seen is seldom or never studied. It is so with pulmonary tuberculosis. It has entered, and still enters, the abode of the physician, steals away his child, his brother, or his sister, his father, mother, companion, and even himself has fallen a victim to its insidious aud flattering visitations. Notwithstanding all this, the man of science is too apt to turn away from it, as from the messenger of death. Too frequently all his bulwarks of defence resolve themselves into this soliloquy—there is a destiny which controls our ends. There was but little known of the disease until Laennec, in 1810, published his invaluable work on tubercular phthisis, and drew a line of demarcation between tubercular and other diseases of the lungs.

Since that time there have been many valuable accessions to our knowledge in this department. But within the last few years have been made the principal improvements in pathological and scientific treatment.

The researches and observations of J. Hughes Bennett, of Edinburgh, are justly considered as constituting an important era in the treatment of this victorious enemy over human life. It is not our intention, at the present time, to enter into a detailed account of the symptoms, course, and minute treatment of this disease, neither to advance new views of its general pathology and treatment, but to sum up the evidence touching its nature and predisposing condition of system, and draw from these the philosophical treatment. In doing so, if we should deviate from high authority and prevalent professional opinions, we only say, "Hear me for my cause."

It is with a feeling of disappointment that the student of medicine reads the text-books on the practice of medicine, and finds that while the symptoms and course of the disease are so accurately described, that so little is said of the peculiarity of system which constitutes the predisposition. Its pathology and treatment are mentioned, but generally accompanied by an acknowledgment of ignorance of the one, and almost worthlessness of the other. It is by understanding, or striving to understand, what is the peculiarity of system which predisposes to tuberculosis, that we may expect to accomplish the much-desired ultimatum of curing, or, what is better, preventing pulmonary phthisis. So far as we know, there are but three—or, more properly, but two—theories advanced which endeavor to explain what the peculiarity of system is in those predisposed. The first is that originally advanced by the German physicians, and subsequently endorsed by others, among whom may be mentioned the high authority of Sir James Clark. By these it was taught that the predisposing cause, or condition of system which constituted the diathesis, was portal congestion.

The second is that of J. Hughes Bennett, of Edinburgh, who believes that the diathesis consists in a want of proper assimilation of the oily portions of food. In his own language, "It is a disease of primary digestion."

The third is that first suggested by Professor Rokitansky, of Vienna, and favorably spoken of by Simon and others. By these it was observed that in this disease there was too rapid arterialization of the blood, and that a venous condition of this fluid appeared to be incompatible with the development of tubercle. Although it is not claimed by the observers of the above fact that the too rapid oxygenization of the blood is the peculiar condition of system which constitutes the diathesis, yet we have been convinced that in this was to be found the peculiarity. The symptoms, pathological changes, and most successful treatment, all point to this as being the distinguishing trait between the strumous system and that of one free from such disposition. Then, as a standing point for a further consideration of the subject, we take the position that the too rapid inbibition of oxygen into the blood, and the consumption or rapid elimination of carbon from the blood, is the starting point of the disease; that this is the agent at work in the human system making tubercles; that this affinity for oxygen and consequent consumption of carbon causes the type of development, impressed upon the system, which constitutes the diathesis, habit, or predisposition to tuberculosis.

We shall not here stop to inquire what are the original anatomical or perceptible differences between such a system and one healthy, but shall be content in summing up the facts which go to prove that such affinities do really exist.

The most eminent pathologists have taught that some hereditary peculiarity distinguished the subjects of phthisis. Indeed, it is said to be pre-eminently hereditary, there being a type of cell-growth given to the offspring by the parent, in accordance with which it is developed; and, as Simon says, it is an imperfect pattern of development. This we can readily understand, in a physiological point of view, in the transmission of family likeness and peculiarity. We know that there is a difference between the cell-growth of man and other animals, and between the different races of man, and also between the different genera and species of animals. We are not surprised to see animals breed after their kind, and even to retain the exact impress of the species and stock of their ancestry. We furthermore expect that they will not only be born, but develop in accordance with this law. In those instances the development is physiological. In the case of those predisposed to this disease, the development—in proportion as the habit prevails—is pathological. Pathological action has always offered more obstacles to the inquirer than physiological. In the subjects of this disease, it would appear that the progeny, the germs of which are first formed in the parents, are developed in accordance with a certain pathological impress given to the first cells.

Simons uses the following language in regard to this hereditary transmission of the disease: "I do not mean that, in the process of impregnation, actual tubercular matter passes from the system of the scrofulous father into the germ of the infant, to remain latent there till circumstances call for its development, nor that, during uterine life, the blood of the child is poisoned by its mother's blood, as occurs in small-pox or syphilis. What I mean is this, that the scrofulous diathesis, that the disposition to form tubercles, is transmitted."

Now, as this tuberculous matter is formed out of the blood, it of necessity follows that it is the result of a change wrought upon that fluid either in the process of digestion and assimilation in the glands of excretion and secretion, or produced by the act of respiration. For the further investigation of these blood changes, it would be important to know what conditions of system favored, and what opposed, such dispositions. We find that there are certain diseases which are incompatible with tuberculosis, among which we will mention that of cancer.

In cancer the oily globules of the blood are in excess, while fibrine is proportionately deficient. There is too much respiratory or carbonaceous material which the system makes no use of, and eventually this kind of cells become so abundant that an artificial excretion is established to relieve the system of this plethora. The patient dies, devoured by parasites in the shape of carboniferous cancer cells, which are continually forming within, and carrying without the body its nutritive material.

We would remark in this connection, that the observation of authors lead us to important truths with regard to the liability of animals to this disease. Commencing with man, it is found that as we descend in the scale to the torpid animals and reptiles, in which a venous state of the blood is prominent, and their consumption of oxygen scant, that the liability to tuberculosis diminishes in the same ratio of descent.

Ague is another disease with which it is rarely associated. There is a marked contrast between the sallow skin and sclerotica of the one, and the fair or natural skin and the bright blue eye of the other; between the mental torpidity and clouds of the one, and the keen, active, unclouded mental perceptions of the other. Ague deranges the liver by overtaxing it, or forcing into it blood containing an excess of carbon, while phthisis is attended by defective bilious secretion because of the blood's poverty in carbon. Bile is carbonaceous, and the liver is strictly an eliminator of carbon. One disease is characterized by its excess, the other by its deficiency.

As we before stated, Rokitansky, whose opinions were derived from actual observation, attached great importance to increased venosity of blood. Under the head of antagonistic conditions to the formation of tubercle, says Simon, he includes every influence which interposes, directly or indirectly, with oxygenization of the blood, either by diminishing the capacity of the chest, or hindering the expansion of the lungs, or by deranging the pulmonary circulation of the blood, or by impeding the free access thereto; for example, a case of spinal deformity, narrowing the chest; a case of abdominal tumor, encroaching upward and causing dyspepsia; a case of cyanosis, maintaining deficient aeration of the blood; these would be cases in which, according to this observer of hundreds of thousands, the tubercular deposit would not be formed.

Among the incompatible diseases is one directly applicable to the point—cyanosis. Chronic venosity of the blood, as it occurs in dependence on congenital malformation of the heart, is called cyanosis; and the blueness of surface from which this class of cases derives its name, depends on the insufficient exhalation of carbonic acid. The heart, owing to its mechanical imperfections, cannot circulate the blood rapidly enough, cannot expose it often enough to the air. Thus the carbonic acid accumulates, and maintains the blood in a venous condition, while at the same time the mal-formed organ, by delaying the systemic circulation, and causing great congestion of the carbonated blood in the general capillary system, renders the unhealthy tint of the blood more apparent to the eye than it would be in any simple case of poisoning by carbonic acid. If the subjects of such diseases delay to die, as sometimes they do, for years, they vegetate with a general torpor and feebleness of vitality, with an incapacity for muscular or even mental exertion, with an extreme susceptibility of fatigue, and with a defective resistance to cold, which sufficiently mark the morbid chemistry of their blood.

There is one signal peculiarity which attends this chronic venous condition of the blood, and not only in extreme cases of cyanosis, but in all chronic diseases where, from any cause whatever, there is defective arterialization of the blood—the patient enjoys one privilege. He is exempt, perhaps not absolutely, but at least all but absolutely, from tubercular disease.

The foregoing conditions of the system in disease, and the exemption which they afford the patient from tuberculosis, are cited as negative proof that the disease depends on the too rapid oxygenization of the blood. We sum up the following as affirmative:

Oxygen is said to be the vivifier, or nature's own stimulant to the system, while carbon is the stupefier, or nature's anodyne to prevent undue excitement. If we breathe carbonic acid we die of asphyxia, or a want of this stimulus. If we breathe oxygen we live too fast, and die soon. Strumous subjects have all the signs of living fast; they are proverbial for precocious minds, and clear, keen perceptions. A quick pulse and excitable nervous system mark the tuberculous, while he whose system is full of carbon is dull, cloudy, and sluggish, both in body and mind.

Oxygen is the special agent in the production of fibrine. There is excess of fibrine in this disease. Carbon is the companion of the oily principle in the blood, hence the deficiency of respiratory material. Upon this assumption, and this alone, can we explain the preference which the tuberculous deposit manifests for the lungs and glandular system. In the lungs the blood undergoes the great change of absorbing oxygen and eliminating carbonic acid, and here we find the favorite seat for this deposit. It is not improbable that the tuberculous matter is precipitated immediately from the blood by the action of oxygen upon its salts and fibrine. The best pathologists consider tubercle altered fibrine. The oxygen enters the lungs, consumes all the respiratory material presented to it by the blood; then having a surplus, the remainder acts upon the fibrine and other constituents of the blood, producing a precipitate, consisting of these matters deprived of vitality by oxygen. This, in its simplest form, is the cacoplastic deposit, known as gray tubercle, which contains considerable animal matter. The calcareous change, or degeneration of tubercle, results from a constant effort to cure the disease by nature, which absorbs the animal portions of the deposit. The source of fat in tubercle is evidently the albuminous compounds, which, like muscle, fibrinous exudations, and blood, may be transformed into oily granules by a chemical process not yet actually determined. The same may be said of fatty degeneration of the liver and other glands which are so frequently observed in phthisical subjects. It would appear that the condition of the system in which fat accumulates in the blood is directly antagonistic to that in which fatty degeneration of its glands takes place. To promote the transformation or absorption of tubercle we must support the strength by nourishing diet, supply respiratory material to the blood, and promote, as far as possible, a free and healthy circulation of the blood in the lungs. Cod-liver oil and iron justly hold a prominent position.

The foregoing is a brief summary of our views as to the general pathology of phthisis.

